# Case Report: Whole-Genome Sequencing of Serially Collected *Haemophilus influenzae* From a Patient With Common Variable Immunodeficiency Reveals Within-Host Evolution of Resistance to Trimethoprim-Sulfamethoxazole and Azithromycin After Prolonged Treatment With These Antibiotics

**DOI:** 10.3389/fcimb.2022.896823

**Published:** 2022-06-01

**Authors:** Paul Christoffer Lindemann, Haima Mylvaganam, Oddvar Oppegaard, Inger Lill Anthonisen, Nermin Zecic, Dagfinn Skaare

**Affiliations:** ^1^ Department of Microbiology, Haukeland University Hospital, Bergen, Norway; ^2^ Department of Medicine, Haukeland University Hospital, Bergen, Norway; ^3^ Department of Microbiology, Vestfold Hospital Trust, Tønsberg, Norway

**Keywords:** *Haemophilus influenzae*, CVID, multidrug resistance, case report, persistence

## Abstract

We report within-host evolution of antibiotic resistance to trimethoprim-sulfamethoxazole and azithromycin in a nontypeable *Haemophilus influenzae* strain from a patient with common variable immunodeficiency (CVID), who received repeated or prolonged treatment with these antibiotics for recurrent respiratory tract infections. Whole-genome sequencing of three longitudinally collected sputum isolates during the period April 2016 to January 2018 revealed persistence of a strain of sequence type 2386. Reduced susceptibility to trimethoprim-sulfamethoxazole in the first two isolates was associated with mutations in genes encoding dihydrofolate reductase (*folA)* and its promotor region, dihydropteroate synthase (*folP*), and thymidylate synthase (*thyA*), while subsequent substitution of a single amino acid in dihydropteroate synthase (G225A) rendered high-level resistance in the third isolate from 2018. Azithromycin co-resistance in this isolate was associated with amino acid substitutions in 50S ribosomal proteins L4 (W59R) and L22 (G91D), possibly aided by a substitution in AcrB (A604E) of the AcrAB efflux pump. All three isolates were resistant to aminopenicillins and cefotaxime due to TEM-1B beta-lactamase and identical alterations in penicillin-binding protein 3. Further resistance development to trimethoprim-sulfamethoxazole and azithromycin resulted in a multidrug-resistant phenotype. Evolution of multidrug resistance due to horizontal gene transfer and/or spontaneous mutations, along with selection of resistant subpopulations is a particular risk in CVID and other patients requiring repeated and prolonged antibiotic treatment or prophylaxis. Such challenging situations call for careful antibiotic stewardship together with supportive and supplementary treatment. We describe the clinical and microbiological course of events in this case report and address the challenges encountered.

## Introduction

Nontypeable *Haemophilus influenzae* (NTHi) frequently colonize the respiratory tract in patients with chronic lung disease or impaired immune system. Bacterial colonization is an independent risk factor for progression to respiratory tract infections, often requiring antibiotic treatment in such patients (Pulvirenti et al., 2018). Recurrent infections and prolonged exposure to antibiotics facilitate development of resistance by increasing the frequency of horizontal acquisition of resistance genes and the rate of adaptive chromosomal resistance mutations and selection of resistant subpopulations (Blazquez et al., 2012; Baquero et al., 2021). With the introduction of whole-genome sequencing (WGS), several examples of within-host resistance development following antibiotic exposure have been described (Didelot et al., 2016; Gatt and Margalit, 2021), but well-documented cases have not been reported in *H. influenzae*, to our best knowledge.

Common variable immunodeficiency (CVID) is the most prevalent symptomatic primary immune disorder and comprises a heterogenous group of clinical conditions characterized by low levels of circulating immunoglobulins and compromised production of specific antibodies, rendering CVID patients particularly vulnerable to respiratory tract infections (Bonilla et al., 2016). Recurrent lung infections impose a long-term risk of development of bronchiectasis and pulmonary sequelae, further fueling the disposition for bacterial infections and the need for antibiotic treatment (Janssen et al., 2021).

Immunoglobulin substitution therapy is the mainstay of CVID management but is not always sufficient to abate recurring infections. Long-term antimicrobial prophylaxis has been advocated as beneficial in selected patients, but clinical trials supporting this practice are scarce (Bonilla et al., 2016), and the risk of development and selection of resistant strains during treatment is insufficiently investigated. We describe a patient with CVID, where long-term treatment with trimethoprim-sulfamethoxazole and azithromycin led to evolution of resistance towards these antibiotics in an *H. influenzae* strain persisting in the respiratory tract during April 2016 - January 2018.

## Case Report

**Figure 1:**
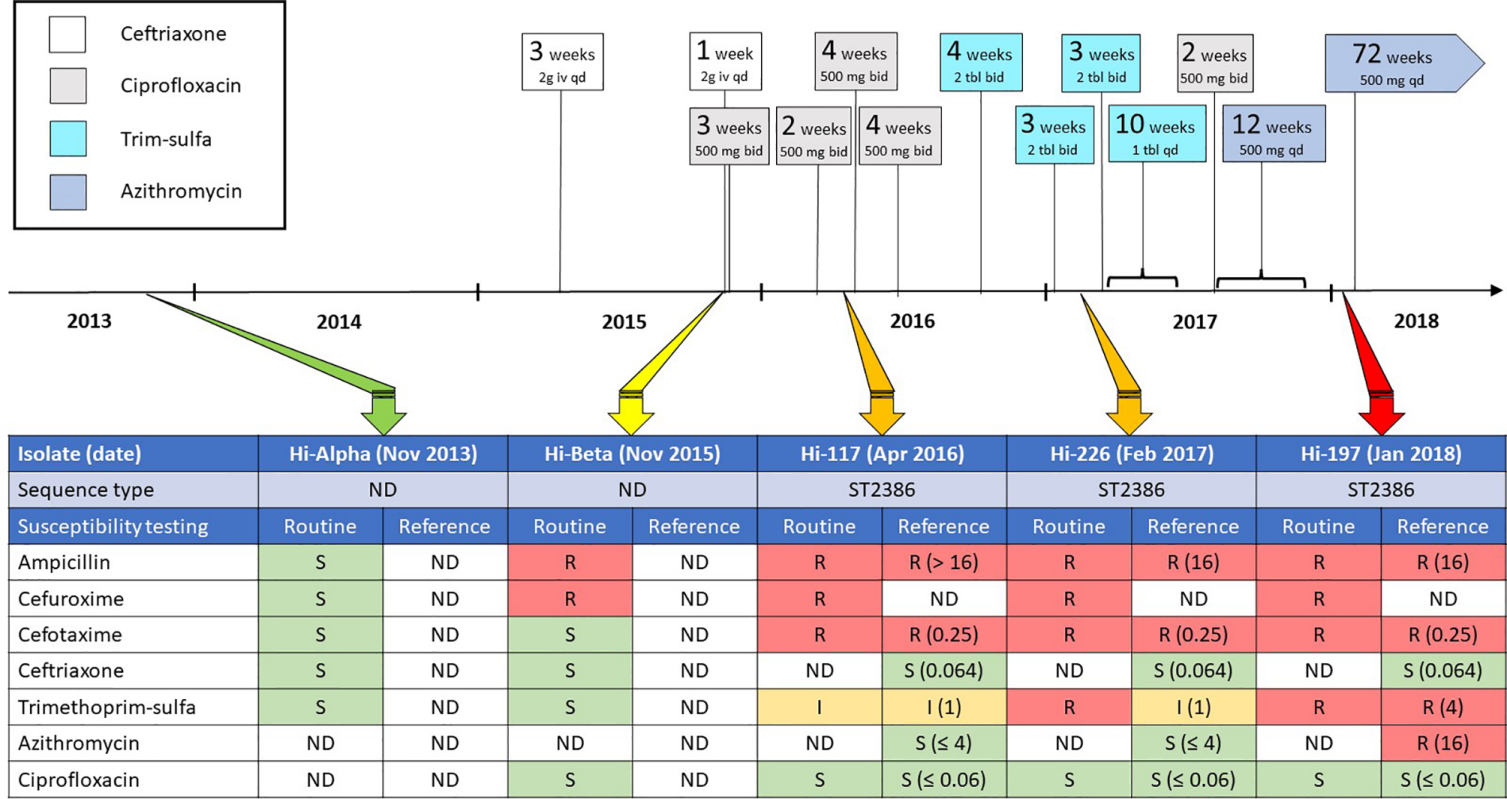
Time scale annotated with exposure to antibiotics (including dosages), along with results from phenotypic antimicrobial susceptibility testing (AST) to relevant antibiotics for five *H. influenzae* isolates sampled between November 2013 and January 2018. The Hi-Alpha and Hi-Beta isolates were not available for genomic characterization, and their potential phylogenetic relationship to the Hi-117, Hi226 and Hi-197 isolates could not be explored. Routine AST results for the five isolates represent primary testing with disk diffusion and/or gradient diffusion, whereas reference AST results were produced retrospectively by determination of broth microdilution (BMD) MIC using custom panels. Disk diffusion and BMD were done according to the standards of the EUCAST, while gradient diffusion was performed according to the manufacturer’s recommendations. AST results were interpreted using EUCAST clinical breakpoints (v. 12.0), except azithromycin (no clinical breakpoints), for which susceptibility categorization was based on the epidemiological cut-off value (4 mg/L). bid, twice daily; qd, once daily; Trim-sulfa, trimethoprim-sulfamethoxazole; S, susceptible; I, intermediately susceptible (changed to “susceptible, increased exposure” from 2019); R, resistant; ND, no data.

A 48-year-old Norwegian male with a history of recurring sinopulmonary infections was admitted to Haukeland University Hospital (Bergen, Norway) in 2011 with pneumonia and concurrent agammaglobulinemia. Following a thorough diagnostic evaluation excluding lymphoid and bone marrow malignancy, chronic viral infections, protein loss and drug-induced adverse reactions, the patient was diagnosed with CVID. Immunoglobulin replacement therapy was initiated in 2012, and by the end of 2013 he achieved sustained IgG levels above 6 g/L. The first isolation of *H. influenzae* from a sputum sample was in November 2013, susceptible to beta-lactam antibiotics and trimethoprim-sulfamethoxazole (Hi-Alpha; **Figure 1**).

Over the course of the next two years, the patient experienced few sinopulmonary infections, but in November 2015 he was admitted to hospital with pneumonia. Microbiological samples were procured by bronchoscopy and *H. influenzae* was identified as sole pathogen (Hi-Beta; **Figure 1**). This isolate displayed resistance to ampicillin and cefuroxime due to beta-lactamase production and mutations in penicillin-binding-protein 3 (PBP3), but was susceptible to cefotaxime, ceftriaxone, trimethoprim-sulfamethoxazole, and ciprofloxacin. The patient received ceftriaxone for five days, followed by three weeks of oral ciprofloxacin.

In March 2016, saline inhalation therapy was commenced by pulmonologists after bronchiectasis was detected on CT scan. *H. influenzae* was again isolated from sputum in April 2016 (Hi-117; **Figure 1**). Hi-117 expressed resistance to ampicillin, cefuroxime, and cefotaxime, and was categorized as intermediately susceptible to trimethoprim-sulfamethoxazole. Four weeks of ciprofloxacin was prescribed.

During the period 2016 to 2018 the patient had IgG trough levels above 8 g/L and received intensified inhalation therapy. Despite this, he had frequently recurring infections, requiring repeated and prolonged courses of antimicrobial therapy. Cultivation of sputum in February 2017 revealed *H. influenzae* (Hi-226) with a resistance pattern similar to Hi-117 from 2016 (**Figure 1**).

Given the deteriorating clinical course and the short-lived improvement with each antibiotic course, a decision was made to attempt long-term antimicrobial prophylaxis. A ten-week course of low-dose trimethoprim-sulfamethoxazole 80 mg/400 mg daily was initiated in March 2017, followed by two weeks of ciprofloxacin 500 mg bid, immediately succeeded by twelve weeks of azithromycin 500 mg daily from September 2017. Rapid amelioration of respiratory symptoms was observed, and the patient returned to a full working status.

In January 2018, he experienced worsening respiratory symptoms, four weeks after terminating prophylactic azithromycin. *H. influenzae* cultivated from sputum (Hi-197, **Figure 1**), was resistant to trimethoprim-sulfamethoxazole. Prophylaxis with azithromycin, 500 mg once daily, was resumed on a permanent basis. Retrospective testing using broth microdilution (BMD) revealed increased MICs to both trimethoprim-sulfamethoxazole and azithromycin in Hi-197, compared to Hi-117 and Hi-226. However, as susceptibility to azithromycin was not tested routinely, this information was not available to influence the choice of antibiotic. Although clinical improvement was observed initially, the efficacy of azithromycin gradually declined during 2018.

By January 2019, his disease burden was like the state prior to initiation of prophylaxis. In September 2019, a bronchoalveolar lavage performed at another hospital revealed profuse growth of *H. influenzae* with a resistance profile identical to that of Hi-197. During 2013-2020, despite comprehensive conventional and molecular investigations, other respiratory pathogens were detected only twice; DNA of *Mycoplasma pneumoniae* in 2016 and influenza B virus RNA in 2018.

His condition rapidly deteriorated and he developed several CVID-complications, including granulomatous lymphocytic interstitial lung disease, liver cirrhosis, and ultimately a high-grade B-cell lymphoma. He died in July 2020 from a neutropenic sepsis caused by *Escherichia coli* and *Staphylococcus aureus*.

## Phenotypic and Genotypic Characterization of *H. influenzae*

**Figure 1** shows a time scale with antibiotic exposure, along with results from phenotypic antimicrobial susceptibility testing (AST) to relevant antibiotics for five *H. influenzae* isolates, identified as the sole pathogen from samples representative for lower respiratory tract, between November 2013 and January 2018. Routine AST results for these five isolates represent primary testing with disk diffusion (Oxoid/Thermo Fisher Scientific, Basingstoke, UK) and/or gradient diffusion (Liofilchem, Roseto degli Abruzzi, Italy), whereas AST for the sequenced strains was also determined by BMD using custom panels (Sensititre NONAG7, Thermo Fisher Scientific). Disk diffusion and BMD were done according to the standards of the European Committee on Antimicrobial Susceptibility Testing (EUCAST), while gradient diffusion was performed according to the manufacturer’s recommendations. AST results were interpreted using EUCAST clinical breakpoints (v. 12.0), except azithromycin, for which susceptibility categorization was based on the epidemiological cut-off value (4 mg/L).

Genetic relationship between Hi-117, Hi-226, and Hi-197 and their molecular basis for resistance development were assessed using WGS (Ion Torrent S5XL, Thermo Fisher Scientific). Trimmed sequencing reads (PHRED score ≥ 20) were analyzed with respect to conventional and core-genome multi-locus sequence typing, ((cg)MLST) with subsequent assignment to Minimum Spanning Tree (MST) clusters (Ridom SeqSphere+ v.8.0). Hi-117, Hi-226, and Hi-197 shared a novel MLST profile (ST2386) comprising a novel *adk* allele (**Table 1**) and belonged to the same MST cluster, separated by 1-7 allele differences in 1589 genes. The results confirm that Hi-117, Hi-226, and Hi-197 represent a single strain, persisting during April 2016 to January 2018.

**Table 1:** Molecular characteristics of Hi-117, Hi-226, and Hi-197.

Parameter		Characteristics		
Strain ID (accession)		Hi-117 (GCA_923276745)	Hi-226 (GCA_923282765)	Hi-197 (GCA_923283335)
Sample type (date)		Sputum (April 2016)	Sputum (February 2017)	Sputum (January 2018)
MLST^a^		ST2386 (254-11-18-18-62-1-5)	ST2386 (254-11-18-18-62-1-5)	ST2386 (254-11-18-18-62-1-5)
cgMLST^b^		MST cluster 9	MST cluster 9	MST cluster 9
Capsular serotype^c^		Nontypeable	Nontypeable	Nontypeable
Other virulence determinants^d^		*hmw*, *hap, igaA1*	*hmw*, *hap, igaA1*	*hmw*, *hap, igaA1*
Transferable resistance genes^e^		*bla*_TEM-1B_	*bla*_TEM-1B_	*bla*_TEM-1B_
Chromosomal resistance^f^				
- Beta-lactams^g^	PBP3 (*ftsI*)	D350N, S357N, M377I, S385T, R517H, T532S, V547I	D350N, S357N, M377I, S385T, R517H, T532S, V547I	D350N, S357N, M377I, S385T, R517H, T532S, V547I
	PBP3 group	High III-like(-)	High III-like(-)	High III-like(-)
- Quinolones^h^	GyrA (*gyrA*)	–	–	–
	ParC (*parC*)	–	–	–
- Azithromycin^i^	L4 (*rpl4*)	–	–	**W59R**
	L22 (*rpl22*)	–	–	**G91D**
	23S rRNA	–	–	–
- Trimethoprim-sulfamethoxazole^j^	DHFR (*folA*)	N13S, W31R, L67P, E69K, I74V, F79L, I95L, K107Q, E135K	N13S, W31R, L67P, E69K, I74V, F79L, I95L, K107Q, E135K	N13S, W31R, L67P, E69K, I74V, F79L, I95L, K107Q, E135K
	DHFR (promoter)	A(-32)C, T(-24)C, G(-4)A	A(-32)C, T(-24)C, G(-4)A	A(-32)C, T(-24)C, G(-4)A
	DHPS (*folP*)	N87S, V95A, V101I, N108S, A150V, I177V, G189C, I210N, I236V, A240V, V268I, A273E	N87S, V95A, V101I, N108S, A150V, I177V, G189C, I210N, I236V, A240V, V268I, A273E	N87S, V95A, V101I, N108S, A150V, I177V, G189C, I210N, **G225A**, I236V, A240V, V268I, A273E
	TS (*thyA*)	H26R, V107I, E238K, T253S	H26R, V107I, E238K, T253S	H26R, V107I, E238K, T253S
- Efflux^k^	AcrR (*acrR*)	S14L, R22K, N26D, Q27R, L31H, L33I, T77S, I121V, H131D, Q134K	S14L, R22K, N26D, Q27R, L31H, L33I, T77S, I121V, H131D, Q134K	S14L, R22K, N26D, Q27R, L31H, L33I, T77S, I121V, H131D, Q134K, **S181F**
	AcrA (*acrA*)	M20I, G32E, M67L, A75T, V76I, V147L, S149N, A156V, D253N, V345A, D369G, I473V	M20I, G32E, M67L, A75T, V76I, V147L, S149N, A156V, D253N, V345A, D369G, I473V	M20I, G32E, M67L, A75T, V76I, V147L, S149N, A156V, D253N, V345A, D369G, I473V
	AcrB (*acrB*)	P660A, T828N, F837Y, A854T, V855T, A858I, I862V, H942Y, V1015I	P660A, T828N, F837Y, A854T, V855T, A858I, I862V, H942Y, V1015I	**A604E**, P660A, T828N, F837Y, A854T, V855T, A858I, I862V, H942Y, V1015I

a Multi-locus sequence typing (MLST) with assignment to sequence types (ST) based on allelic profiles of seven housekeeping genes (adk, atpG, frdB, fucK, mdh, pgi, and recA). ST2386 is a single-locus variant (SLV) of ST836 with the novel allele adk-254 (Jolley et al., 2018).

b Core genome MLST (cgMLST) with assignment to Minimum Spanning Tree (MST) cluster was performed with Ridom SeqSphere+ v. 8.0 (Münster, Germany) on a collection of 222 clinical isolates of H. influenzae from Norway or Sweden (BioProject PRJEB49398).

c Capsular serotyping was performed with Hicap v.1.0.3 (Watts and Holt, 2019).

d Virulence determinants were called using a locally installed version of MyDbFinder v.2.0 with a custom database comprising genes from the virulence factor database (VFDB) (Liu et al., 2022) and the following additional sequences, database downloaded 2021-08-24: hmwA1 (first 1269 bp) (NZ_LN831035.1), hap (U11024.1), hia (U38617.2), igaA2 (NDZN01000054.1), igaB1 (DQ423203), and igaB2 (KC607498). Thresholds of 60% were used for identity and coverage.

e Transferable resistance genes were called with ResFinder v.4.1 (Bortolaia et al., 2020), using thresholds of 60% for identity and coverage. bla_TEM-1B_, 100% identity and coverage (AY458016).

f Alterations in chromosomally encoded proteins, genes (in brackets) or promoter regions were called by multiple sequence alignment of translated coding genes using the msa package for R (Bodenhofer et al., 2015) and H. influenzae Rd KW20 (GCA_000027305.1) as reference. Amino acid substitutions were confirmed by mapping of quality-trimmed sequencing reads (PHRED score ≥ 20) against the reference sequence using BWA (Li, 2013), with subsequent variant calling and annotation using FreeBayes (Garrison and Marth, 2012) and SnpEFF (Cingolani et al., 2012).

g Substitutions in penicillin-binding protein 3 (PBP3) (transpeptidase region, aa 327-610) and grouping according to Skaare et al., 2014 (Skaare et al., 2014).

h Substitutions in DNA gyrase (GyrA, subunit A) or DNA topoisomerase IV (ParC, subunit A) (quinolone-resistance determining regions, QRDR; aa 80-92) (Georgiou et al., 1996).

i Substitutions in 50S ribosomal proteins L4 or L22, or single nucleotide polymorphisms (SNPs) in the six copies of the 23S rRNA gene (rrnA23S-rrnR23S) (peptidyl transferase center, nt 1900-2520) (Fyfe et al., 2016).

j Substitutions in dihydrofolate reductase (DHFR) (or SNPs in promoter region), dihydropteroate synthase (DHPS), or thymidylate synthase (TS) (Fernandez-Villa et al., 2019).

k Alterations in the operon encoding and regulating the AcrAB efflux pump.

Differences between strains in bold.

**Table 1** summarizes the molecular basis of resistance to the different antibiotic groups, and depicts virulence determinants that might influence persistence, despite antibiotic therapy. Hi-117, Hi-226, and Hi-197 had *bla*_TEM-1B_ and identical amino acid substitution patterns in PBP3, including the S385T substitution associated with high-level beta-lactam resistance (Skaare et al., 2014). They also shared multiple amino acid substitutions in enzymes involved in the folate pathway; dihydrofolate reductase (DHFR), dihydropteroate synthase (DHPS), and thymidylate synthase (TS) (Fernandez-Villa et al., 2019), in addition to single nucleotide polymorphisms (SNPs) in the DHFR promoter region. Variant calling (see **Table 1** for methodology) showed that Hi-197, which expressed significantly higher MICs to trimethoprim-sulfamethoxazole and azithromycin compared to Hi-117 and Hi-226 (**Figure 1**), possessed additional substitutions in DHPS (G225A), 50S ribosomal proteins L4 (W59R) and L22 (G91D), and the components AcrR (S181F) and AcrB (A604E) of the AcrAB efflux pump. All these mutations had 100% allelic uniformity and convincing depths of coverage (range 41-74). The macrolide binding site of 23S rRNA (Fyfe et al., 2016) and the quinolone-resistance determining regions of GyrA and ParC (Georgiou et al., 1996) lacked resistance-conferring mutations. *bla*_TEM-1B_ was the only horizontally acquired resistance gene.

## Discussion

This study describes persistent colonization of the respiratory tract with an NTHi strain despite antibiotic treatment in line with contemporary CVID guidelines, complicated by within-host evolution of resistance-conferring mutations and selection of subpopulations resistant to trimethoprim-sulfamethoxazole and azithromycin, during long-term prophylaxis with these antibiotics.

To our best knowledge, this is the first well-documented example of within-host evolution of antibiotic resistance in *H. influenzae*. Pfeifer et al. reported the emergence of a multidrug-resistant *H. influenzae* strain in a CVID patient but were unable to demonstrate persistence because earlier strains were not available for molecular characterization (Pfeifer et al., 2013). A study conducting partial genomic analyses on serially collected NTHi from a bronchiectasis patient revealed genetic changes associated with resistance to antimicrobial peptides in a persistent strain (Garmendia et al., 2014). Two later studies applied WGS for investigation of genetic changes associated with host adaption during persistent respiratory tract infection with NTHi, but within-host evolution of antibiotic resistance was not demonstrated (Moleres et al., 2018; Pettigrew et al., 2018).

**Figure 2:**
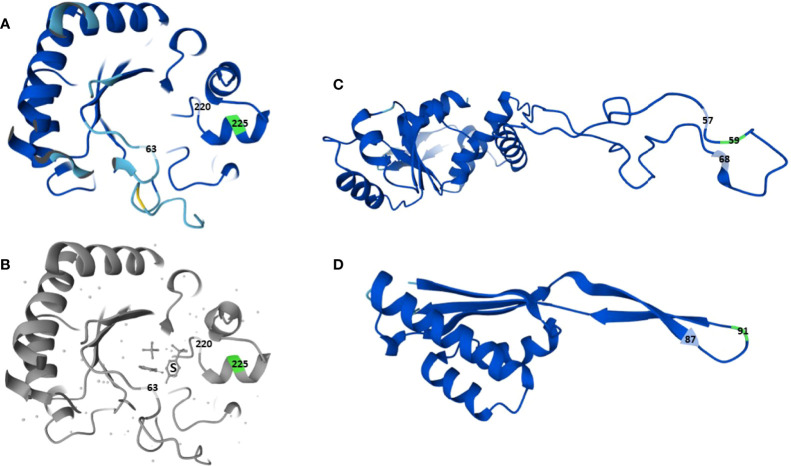
Three-dimensional structure of dihydropteroate synthase (DHPS) and 50S ribosomal proteins L4 and L22 in *H. influenzae* Rd KW20, with positions of newly acquired substitutions in strain Hi-197 highlighted (green). **(A** top left**)** DHPS in Rd KW20 (P43776), with focus on the binding site for sulfonamides. **(B** bottom left**)**, DHPS in *E. coli* K12 (P0AC13) with the sulfonamide molecule (S) sandwiched between amino acid positions 63 and 220. **(C, D** right**)**, 50S ribosomal proteins L4 [P44345, **(C** top**)**] and L22 [P44360, **(D** bottom**)**] in Rd KW20, with focus on the highly conserved regions _57_KPWRQKGTGRAR_68_ (L4) and _87_PRAKG_91_ (L22) of the extended hairpin loops. Screenshots from UniProt (UniProt, 2021), licenced under CC BY 4.0.

Trimethoprim interferes with the folate pathway by inhibiting the enzyme DHFR (encoded by *folA*), and sulfamethoxazole by inhibiting DHPS (encoded by *folP*) (Fernandez-Villa et al., 2019). Resistance to trimethoprim-sulfamethoxazole in *H. influenzae* is usually due to target alterations caused by *folA* or *folP* mutations, DHFR overexpression due to mutations in the *folA* promoter region, thymidine auxothrophy because of loss-of-function mutations in *thyA*, or horizontally acquired *sul* genes encoding sulfonamide-resistant isoforms of DHPS (Enne et al., 2002; Rodriguez-Arce et al., 2017; Sierra et al., 2020). The low-level resistant Hi-117 and Hi-226 harbored several substitutions in DHFR and DHPS, including three SNPs in the DHFR promoter region (**Table1**), previously described in resistant *H. influenzae* (Enne et al., 2002; Sierra et al., 2020). The additional DHPS substitution G225A in Hi-197 is to our best knowledge novel. Crystallography shows that sulfonamides are sandwiched between amino acids 63 and 220 in DHPS in *E. coli* (Achari et al., 1997). The proximity of position 225 to the sulfonamide binding site (**Figure 2**) and the lack of other changes in relevant genes make it plausible that G225A caused the significant increase in trimethoprim-sulfamethoxazole MIC in Hi-197.

Azithromycin inhibits protein synthesis in *H. influenzae* through dual inhibitory effects on synthesis and function of the 50S ribosomal subunit (Champney and Miller, 2002). The drug binds to 23S rRNA (nt 2058-2059) downstream of the peptidyl transferase center, blocking the peptide exit tunnel close to a constriction formed by hairpin loops of ribosomal proteins L4 and L22 (Fyfe et al., 2016). Azithromycin resistance in *H. influenzae* is predominantly caused by chromosomal mechanism such as target alterations due to 23s rRNA mutations or altered ribosome-macrolide interaction due to L4 and/or L22 substitutions; however, rare strains with efflux-mediated resistance encoded by horizontally acquired *mel* and *mef* genes have been reported (Atkinson et al., 2017). Hi-197 contained substitutions G91D in L22 and W59R in L4. While G91D has been described in macrolide-resistant *H. influenzae*, W59R seems novel and is located within the highly conserved region _57_KPWRQKGTGRAR_68_ of the extended hairpin loop of L4 (**Figure 2**), where alterations are associated with macrolide resistance (Fyfe et al., 2016). This strongly suggests that both substitutions contributed to the azithromycin-resistant phenotype of Hi-197. In addition, while Hi-117, Hi-226 and Hi-197 shared several substitutions in AcrA, AcrB, and the repressor AcrR of the AcrAB efflux pump, the azithromycin resistant Hi-197 had additional substitutions in AcrB (A604E) and AcrR (S181F). Stepwise resistance to azithromycin has been reported with truncated AcrR and a subsequent substitution in AcrB (R327S) (Seyama et al., 2017). Truncated AcrR was not found in our strain, but like R327S, the A604E substitution is in the periplasmic domain where ligand binding occurs (Daley et al., 2005). Accordingly, we cannot exclude that the AcrB substitution might have contributed to azithromycin resistance; however, as Hi-197 expressed wild-type susceptibility to quinolones, chloramphenicol, tetracyclines, gentamicin, and rifampicin (data not shown), the effect of A604E might be discrete and/or azithromycin selective.

Whole-genome phylogenetic analysis revealed persistence of an ST2386 *H. influenzae* strain during April 2016 - January 2018, despite repeated and prolonged exposure to antibiotics.

Evading antibiotic activity by biofilm formation and intracellular invasion of host cells appear to be pivotal mechanisms promoting bacterial persistence (Clementi and Murphy, 2011). Beta-lactams and trimethoprim-sulfamethoxazole penetrate NTHi biofilm poorly. In an *in vitro* study, amoxicillin/clavulanic acid eliminated 100% of planktonic NTHi isolates, but only 3.6% of isolates in biofilm (Slinger et al., 2006). Trimethoprim-sulfamethoxazole eliminated 68% of planktonic but not biofilm isolates. Ciprofloxacin and azithromycin eliminated 100% of planktonic isolates and had biofilm elimination rates of 68% and 57%, respectively.

A notable observation in the present study was that repeated and prolonged courses with ciprofloxacin did not eradicate the strain, despite wild-type susceptibility to ciprofloxacin. This is consistent with the suboptimal abilities of ciprofloxacin to eliminate NTHi in biofilm (Slinger et al., 2006; Cavaliere et al., 2014). As quinolones increase the genome-wide spontaneous mutation rate (Long et al., 2016; Song et al., 2016) and may promote mutational resistance to other drugs (Tanimoto et al., 2008; Didier et al., 2011; Song et al., 2016), an intriguing yet unresolved question is whether ciprofloxacin exposure immediately before azithromycin prophylaxis from September 2017 contributed to the emergence of L4, L22, and AcrR substitutions in Hi-197.

Azithromycin is an appealing choice in the treatment of NTHi infections based on high intracellular activity, a biofilm-penetrating ability superior to most other drugs (albeit far from 100%), and a proven inhibitory effect against biofilm formation (Lode et al., 1996; Slinger et al., 2006; Starner et al., 2008). Moreover, several experimental studies suggest an immunomodulatory effect of azithromycin on host inflammatory response, reducing mucus production and ameliorating chronic inflammation (Parnham et al., 2014). Consequently, azithromycin prophylaxis has gained momentum in the management of chronic and recurrent lung infections.

Contemporary CVID guidelines advocate the use of long-term antibiotics in selected patients, although criteria for identifying such patients are lacking (Bonilla et al., 2016; Polverino et al., 2017; Hanitsch et al., 2020). The use of antimicrobial prophylaxis is nevertheless widespread, administered to 20% - 65% of CVID patients in different temporal and geographic settings, and azithromycin is by far the most frequently prescribed antibiotic (Kuruvilla and de la Morena, 2013; Sperlich et al., 2018). The recommendations and clinical practice predominantly lend support from three randomized controlled trials exploring macrolide prophylaxis for six to twelve months in non-cystic fibrosis bronchiectasis patients (Wong et al., 2012; Altenburg et al., 2013; Serisier et al., 2013). All demonstrated reduced number of infectious exacerbations in the macrolide group.

However, the risk of macrolide resistance is not negligible. In one study, macrolide resistance in pathogens isolated from the respiratory tract increased from 35% to 88% in the azithromycin group during the twelve-month study period, compared to 28% and 25% in the placebo group (Altenburg et al., 2013). Interestingly, emergence of macrolide resistance was not associated with loss of efficacy in the subsequent months, suggesting a pivotal role of the anti-inflammatory properties of azithromycin. This may also have been the case in our patient; however, azithromycin gradually lost activity after twelve months, highlighting the need for publications with long-term follow up.

EUCAST state that there is conflicting clinical evidence for the efficacy of macrolides in *H. influenzae* respiratory infections and have removed the clinical breakpoints (EUCAST, 2017). The Clinical and Laboratory Standards Institute (CLSI) state that susceptibility testing of azithromycin is often not necessary for management of individual patients and categorize the drug as Group C (alternative or supplemental agents) (CLSI, 2021). Accordingly, azithromycin susceptibility was not investigated routinely in the presented case. Retrospective testing revealed that azithromycin prophylaxis was continued for 18 months after the emergence of high-level resistance, exemplifying that discrepancy between clinical and laboratory guidelines might lead to non-beneficial or even potentially harmful treatment. Considering the widespread use of macrolide prophylaxis in CVID and bronchiectasis patients, EUCAST and CLSI recommendations for azithromycin susceptibility testing of *H. influenzae* from these patient groups should be revisited, with more emphasis on harmonization of clinical and laboratory guidelines. Moreover, this case report underlines the importance of maintaining close collaboration between clinicians and microbiology laboratories.

The presented case illustrates that the immunologic impairment in CVID patients extends beyond reduced IgG levels, and they remain at risk of infections despite aggressive IgG substitution therapy. Highly variable titers of antibodies specific to *Streptococcus pneumoniae* and *H. influenzae* in immunoglobulin replacement products, reduced half-life of infused immunoglobulins, and lack of mucosal immunity all likely contribute to the persistent susceptibility to these pathogens (Mooney et al., 2017). Restoration of mucosal immunity is likely not feasible through traditional immunoglobulin substitution therapy. Recent studies with nebulized administration of immunoglobulins or local immunotherapy by sublingual administration of inactivated bacterial pathogens report promising effects on mucosal immunity (Vonarburg et al., 2019; Guevara-Hoyer et al., 2020). However, their clinical efficacy is yet to be evaluated in larger trials. Importantly, the repertoire of virulence determinants in *H. influenzae* includes IgA proteases, as illustrated by the presence of an *igaA1* gene in our strain (**Table 1**).

Reduced vaccine response is one of the diagnostic criteria for CVID and the therapeutic potential of active immunization in this patient group remains questionable. Moreover, no vaccine is available for NTHi, which currently constitute the major concern in CVID patients (Slack et al., 2021).

Biofilm inhibitors have attracted interest as possible supplements to antibiotic therapy or prophylaxis in patients with chronic NTHi infections. DNase I, a mucolytic approved for human use often combined with inhaled antibiotics in cystic fibrosis patients (Manos, 2021), destabilizes biofilm matrix and enhances the efficacy of antibiotic treatment of NTHi biofilms *in vitro* (Izano et al., 2009; Cavaliere et al., 2014). Clarification is needed as to whether DNase I, or other biofilm inhibitors or mucolytic supplements represent useful adjuvants in bronchiectasis and CVID patients.

A weakness of this study is that isolates from 2013 (Hi-Alpha) and 2015 (Hi-Beta) were not available for retrospective characterization, and we had incomplete information about antibiotics prescribed by the patient’s general practitioner during this period. Assessment with respect to development of beta-lactam resistance and duration of persistence prior to 2016 was therefore not possible. Moreover, it is difficult to determine the relative contribution of the persistent *H. influenzae* strain to the patient's deteriorating clinical course after January 2018, especially in the late stages with progressive interstitial lung disease (Granulomatous and Lymphocytic Interstitial Lung Disease (GLILD)). However, we did not detect any other likely causative pathogens in respiratory samples in this period.

## Conclusion

We describe chromosomal mutations responsible for within-host evolution of resistance to trimethoprim-sulfamethoxazole and azithromycin in a persisting NTHi strain in the respiratory tract of a CVID patient, after prolonged exposure to these antibiotics. The presented case illustrates the precariousness of long-term antimicrobial prophylaxis in immunocompromised patients, and the current clinical management and guidelines for CVID should be scrutinized. We highlight the need for a multi-targeted approach for treatment of respiratory tract infections and measures to hinder pathogen persistence and development of antibiotic resistance in this challenging patient group.

## Data Availability Statement

The datasets presented in this study can be found in online repositories. The names of the repository/repositories and accession number(s) can be found below: https://www.ebi.ac.uk/ena, accession numbers: GCA_923276745, GCA_923282765, GCA_923283335.

## Ethics Statement

The use of personal data in this study was approved by the Regional Committees for Medical and Health Research Ethics in Norway (reference number 2018/1558) and the Norwegian Data Protection Services (reference number 232381). The patient’s spouse provided written informed consent to the use of patient data in this study.

## Author Contributions

The idea of the case report was conceived by DS, PL, and HM. PL, HM, OO, and DS wrote the paper. OO is the infectious disease specialist involved in treatment of the patient. The bacterial isolates were obtained and primarily investigated at the laboratory of PL and HM. IA, NZ, and DS performed the reference analyses. All the authors contributed to the revision of the manuscript and approved the final version.

## Funding

This work is funded by a research grant to DS from the South-Eastern Norway Regional Health Authority (project# 2016132).

## Conflict of Interest

The authors declare that the research was conducted in the absence of any commercial or financial relationships that could be construed as a potential conflict of interest.

## Publisher’s Note

All claims expressed in this article are solely those of the authors and do not necessarily represent those of their affiliated organizations, or those of the publisher, the editors and the reviewers. Any product that may be evaluated in this article, or claim that may be made by its manufacturer, is not guaranteed or endorsed by the publisher.
